# The Temporal and Spatial Epidemiology Employed in the Elimination of the HIV Epidemic in the Largest Capital of the Brazilian Rainforest

**DOI:** 10.3390/tropicalmed7090225

**Published:** 2022-09-02

**Authors:** Bruna Rafaela Leite Dias, Taymara Barbosa Rodrigues, Dulce Gomes, Ricardo Alexandre Arcêncio, Elucir Gir, Glenda Roberta Oliveira Naiff Ferreira, Sandra Helena Isse Polaro, Eliã Pinheiro Botelho

**Affiliations:** 1 Graduate Nursing Program, Federal University of Pará, Belem 66075-110, Brazil; 2 Department of Mathematics, Luís António Verney College, University of Évora, 7000-671 Evora, Portugal; 3 Department of Maternal-Infant and Public Health Nursing, College of Nursing at Ribeirao Preto, University of São Paulo, Ribeirão Preto 14040-902, Brazil

**Keywords:** social determinants of health, epidemics, HIV, acquired immunodeficiency syndrome, spatial analysis, time series analysis

## Abstract

Background: The main goal of this study was to analyze the human immunodeficiency virus (HIV) epidemic temporally and spatially in Belém from 2007 to 2018. Methods: The incidence rates were analyzed according to time using autoregressive integrated moving-average models, as well as spatially using spatial autocorrelation, Kernel density, scan statistics, and regression techniques. Results: During the study period, 6007 notifications of new cases of HIV/AIDS were reported. The time series analysis revealed a stabilized trend of incidence from 2007 to October 2016, followed by irregular fluctuations until the end of December 2018. Seasonal behavior was observed from 2019 to 2022. The high–high incidence clusters were found in the central and transition areas. An expansion of the number of new reported cases was observed in the central area. Three spatial risk zones were observed. The higher relative risk zone was concentrated in the transition area. The spatial regression showed that the incidence rates were positively correlated with the Family Health Strategy (FHS) coverage. Conclusions: To eliminate HIV in Belém, it will be necessary to decentralize testing and ART and expand the coverage of FHS to ensure universal access to healthcare for citizens.

## 1. Introduction

The human immunodeficiency virus (HIV) epidemic remains a challenge to global public health. Around the world, about 38 million people are living with HIV (PLHIV). Although universal access to antiretroviral therapy (ART) has reduced the number of new HIV infections [1] worldwide, in Latin America, infections have increased by 21% since 2010 [2]. In 2014, Brazil implemented the UNAIDS policy ‘Treatment for All’ in response to HIV. Since then, a 17.2% reduction in HIV detection rates has been observed. However, this reduction was in the South, Southeast, and Central-West Brazilian regions; in the North and Northeast regions, the HIV detection rate increased by 24.4% and 11.3%, respectively. Belém, the capital of Pará, is the second Brazilian capital and has the highest HIV/AIDS detection rate, as well as the highest AIDS mortality rate [3].

The HIV epidemic is dynamic and directly influenced in space and time by socioeconomic and political territorial characteristics. Therefore, to better understand the epidemic, the temporal and spatial analysis techniques should be applied [4]. In Libya, these techniques were applied to study HIV infections for the identification of areas of higher epidemic prevalence and the behavior of rates during the study period. However, studies using both techniques are still rare [5,6,7]. In a literature review of studies on the incidence of HIV infection and AIDS that used spatial analysis methods, only 14 studies were identified [4,5,6,7,8,9,10,11,12,13,14,15,16,17]. Of these, none employed the geographically weighted regression (GWR) to analyze the influence of social determinants of health (SDH) or the Kernel density to study the spread of the epidemic. In addition, only two studies [5,6] used the temporal approach focusing only on the annual variation of incidence rates. Furthermore, although in the spatial analysis it is possible to analyze the time–space association, the results are restricted to a specific time period, overlooking other important components of the time series, such as seasonality, inflection point, and forecasting. Seasonality analysis is essential to observe whether the detection rate fluctuates, and the inflection point shows the exact time when the behavior of the time series changes. Theses analyses can suggest social or political influences on the epidemic. Forecasting analysis is an important tool to predict how an epidemic will behave in the future and can alert the health authorities to implement public policies in advance.

Therefore, in this study, we analyzed the HIV epidemic in Belém using the Box and Jenkins method to analyze the time series seasonality, the inflection point, and forecasting. For the spatial analysis, we employed the HIV/AIDS detection rate distribution and spatial autocorrelation to identify hotspots for HIV/AIDS incidence, Kernel density to observe the expansion of the HIV epidemic, scan statistics to search for the HIV-risk areas, and GWR to analyze the association of incidence with the SDH. The SDH were chosen based on a literature review showing their influence on the HIV epidemic.

## 2. Materials and Methods

**Figure 1:**
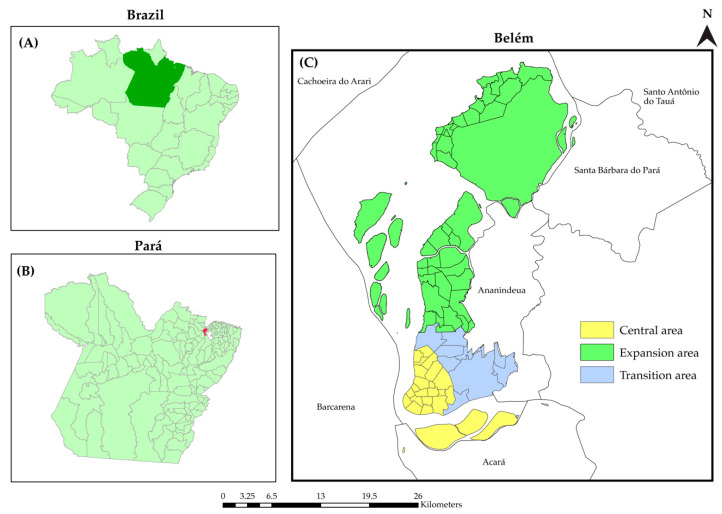
Spatial location of Pará (**A**), Belém (**B**, **red polygon**), and the political map of Belém (**C**). Belém, Pará, Brazil (2020).

This was an ecological study that used a multi-method analysis approach to study the HIV epidemic in Belém using secondary data of new cases of HIV infections and AIDS reported to the Notifiable Diseases Information System (SINAN) in 2007–2018. Belém, the capital of Pará (Figure 1), is the largest city in the Brazilian Amazon region, with a territorial area of 1,059,458 km² and a population of 1,446,042 people [18] living in its 71 neighborhoods and 26 islands [19]. It is the sixth Brazilian municipality and has the highest social vulnerability index (SVI) of 0.317. Moreover, it has a high income inequality Gini index of 0.59 [20,21]. Furthermore, Belém has the highest population density in the Brazilian Amazon region, a low coverage of primary healthcare services (22%), and only two specialized healthcare locations that can address PLHIV.

### 2.1. Unit of Analysis

The cartographic base of the human development unit (HDU), which is publicly available on the website of the Atlas of Human Development [20], was used for the spatial analysis. Belém is divided into 161 HDUs, but we used only 151 units for the analysis. All islands (10 HDUs) were excluded due to the pressure and spatial continuity of the spatial analysis techniques used for the object under study [22].

Of the analysis units, 57 formed the central area of the city, 39 formed the transition area, and 55 formed the expanding area. The central area is the region with the highest concentration of urban services and healthcare locations; it is the region with the highest quality of life in Belém. The transition and expanding areas are characterized by numerous socioenvironmental problems, reflecting the scarcity of public policies [23].

### 2.2. Study Population

The study population was composed of new reported cases of HIV/AIDS in people aged 11 years and older. These data were reported to SINAN and were provided by the Pará Health Department. We included all the notifications that contained the complete home addresses of the cases. Notifications of people living in penitentiaries, asylums or orphanages were excluded. Moreover, the participants were not identified since the data were de-identified.

### 2.3. Study Variables and Data Source

The HIV/AIDS incidence rate was considered a dependent variable. The social and economic indicators of each HDU in the municipality were considered as independent variables. The incidence rate was calculated based on the number of new HIV/AIDS reported cases; the specific population of each HDU was used as the base. Then, the results were multiplied by 100,000 inhabitants.

The demographic data were obtained from the 2010 demographic census of the Brazilian Institute of Geography and Statistics (IBGE), the social and economic data in the Atlas of Social Vulnerability (http://ivs.ipea.gov.br/index.php/pt/; accessed on 1 July 2020), and the Atlas of Human Development (http://www.atlasbrasil.org.br/; accessed on 1 July 2020).

### 2.4. Time Series Analysis

The time series analysis was carried out using the autoregressive integrated moving average (ARIMA), followed by the Box and Jenkins method for data adjustment [24], in RStudio software, version 1.3.959 (RStudio, Boston, MA, USA). We used the monthly HIV/AIDS incidence rates.

The ARIMA model for the time series was estimated by the auto.arima function, which is available in the forecast package. The tests of absence of autocorrelation (Box–Pierce), normality (Kolmogorov–Smirnov), and the *t*-test of average nullity were used to validate the model in the residual analysis. In the graphical analysis, the fit of the forecasts proved to be unsatisfactory, and the seasonal and trend decomposition using the Loess forecasting method was applied. This method combines the STL methodology with ARIMA models to try to replicate patterns for the future [25].

To evaluate the predictive performance of the models, we considered the root mean square error (RMSE), mean absolute error (MAE), and mean absolute percentage error (MAPE). After selection of the best fitting model, the predictive analysis was performed for a 4-year period (2019–2022).

### 2.5. Spatial Analysis

Initially, all the home addresses were georeferenced and geocoded in the QGIS^®^ 2.18 software (OSGeo, Beaverton, OR, USA) for the spatial analysis using the geographic coordinate system decimal degrees for latitude and longitude. The sex-adjusted incidence rates for the 4-year period were calculated for the HDUs to avoid the annual variances (2007–2010, 2011–2014, and 2015–2018), and for the 12 years of the study for each of the HDUs.

The sex-adjusted HIV/AIDS incidence rates for the HDUs were obtained using the direct method [26] in the Microsoft Office Excel 2016 software (Microsoft, Redmond, WA, USA). Then, the Shapiro–Wilk test was applied to verify the normality of the incidence rate distribution.

#### 2.5.1. Spatial Distribution of HIV/AIDS Incidence

The spatial distribution of HIV/AIDS incidence was analyzed through the choropleth maps constructed by ArcGis 10.2 (ESRI, Redlands, CA, USA). We applied the autocorrelation spatial analysis using the Getis–Ord General G with 999 permutations and Getis–Ord Gi*. The first analysis showed the presence or absence of spatial autocorrelation. The second analysis showed the hotspot and coldspot areas. We adopted the first-order queen type matrix and only the municipalities that shared edges and corners were considered as neighbors. Moreover, we considered only clusters with z-scores higher than 1.96 (*p*-value ≤ 0.05).

#### 2.5.2. Kernel Density Estimation and Scan Statistics

The Kernel density estimation technique was used to show the expansion of the HIV epidemic in Belém during the period of the study. It is an exploratory interpolation technique that produces a density surface. The results are shown on a color scale. The increased color intensity indicates a more concentrated event in space [27]. A radius of 1000 m [28] and a quadratic function were adopted as parameters. The analysis was performed using the QGIS^®^ 2.18 software.

To search for the HIV-risk areas, we used the spatial scanning statistical analysis [29] using the SaTScan™ 9.6 software (Martin Kulldorff, Boston, MA, USA). We identified the spatial and space–time clusters using the discrete Poisson model. To calculate the spatial risk, the following criteria were applied: Circular-shaped clusters, clusters with no geographic overlapping, maximum cluster size equal to 50% of the exposed population, and 999 replications. For the spatiotemporal risk analysis, the same criteria were used, except for the maximum temporal cluster size, which was considered to be 50% of the study period. A cluster was considered at risk if its relative risk (RR) was equal to or above 1.0 at a *p*-value of ≤ 0.05. The 95% confidence intervals were estimated in R software using the methods of a previous study [30], and thematic maps were then obtained using ArcGis^®^ 10.2 software.

#### 2.5.3. Spatial Regression Analysis

This analysis was carried out in accordance with the approaches used in a previous study [31]. First, the collinearity between the dependent and independent variables was tested according to Pearson’s correlation in IBM SPSS^®^ software (IBM, Armonk, NY, USA). The variables with significant correlations (*p* < 0.05) were analyzed through the ordinary least square (OLS) regression model. The OLS model with lower multicollinearity was inserted into global (spatial error and spatial lag) models of spatial regression. The best explanatory model was with the higher R^2^, higher adjusted R^2^, and smaller Akaike information criterion (AIC) and *p* < 0.05. After the spatial dependency of the residuals of the chosen model was discarded, the model was evaluated in the GWR, where the bandwidth of the kernel type was chosen based on the lower corrected AIC (AICc). The R^2^, adjusted R^2^, AIC, and AICc values were used to compare the OLS and GWR models. These analyses were performed using Geoda 1.14.0 (Luc Anselin, Chicago, IL, USA) and ArcGis^®^ 10.6 software.

### 2.6. Ethics

The study project was approved by the Research Ethics Committee (CEP) of the Institute of Health Sciences of the Federal University of Pará (ICS/UFPA), under opinion no. 3.331.577. No consent terms were signed since only secondary data were used. Moreover, the participants were not identified.

## 3. Results

### 3.1. Time Series Analysis

During the period of the study, 6007 new cases of HIV/AIDS were reported to SINAN. The majority of the study population was composed of individuals aged 30–49 years old (50.9%), with a combination of men (70.7%), brown color (69.6%), high school level (32.2%), and heterosexual (52.4%) (Table S1). The time series analysis revealed an average of one case per 100,000 inhabitants (inhab.) and a standard deviation of 1.25. The lowest and highest HIV/AIDS incidence rates (0.2 and 8.38) occurred in October 2013 and December 2016, respectively. From 2007 to October 2016, the HIV/AIDS incidence rates presented a stable trend, and an upward trend was observed from November to December 2016. From January 2017, the incidence rate showed irregular upward and downward fluctuations until the end of December 2018. The absence of seasonality was confirmed by the Kruskal–Wallis test with a *p*-value < 0.00.

The best model to describe the variability of the data over time was STL + ARIMA (2,1,2). Table 1 shows the residuals and forecasting parameters of the time series.

**Figure 2:**
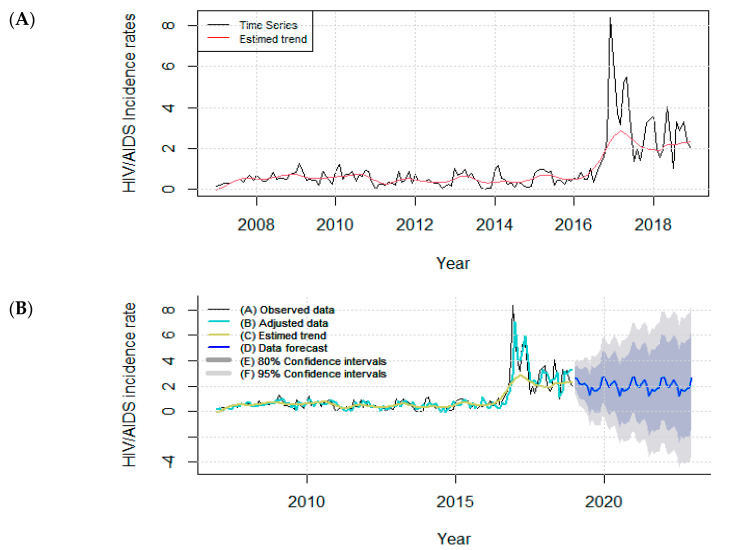
(**A**) Time series of the incidence rates (per 100,000 inhab.) of HIV infection and AIDS in Belém, PA, Brazil (2007–2018). (**B**) The STL + ARIMA (2,1,2) model adjusted to the transformed HIV/AIDS incidence rates (per 100,000 inhab.) and the forecast for Belém, PA, Brazil (2019–2022).

Figure 2A shows the incidence rates from 2007 to 2018. The STL + ARIMA (2,1,2) model was fitted to the HIV/AIDS incidence rates in Belém, PA, and the forecast of these rates from 2019 to 2022 is shown in Figure 2B. Stability in average terms was observed for the forecast period. The highest values were obtained from January to May, followed by a reduction from June to July, when the lowest values were obtained. A small increase was observed in August, followed by a reduction in September, after which the incidence rate gradually increased again.

### 3.2. Spatial Analysis

**Figure 3:**
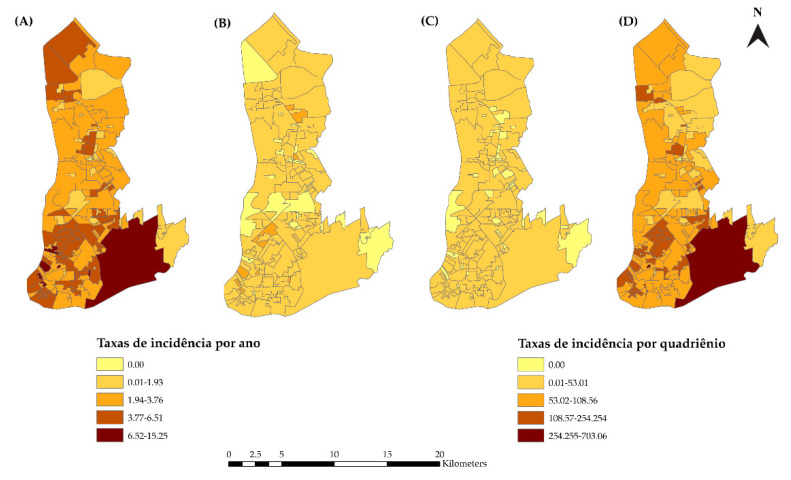
Map of the distribution of the standardized incidence rates of HIV infection and AIDS. (**A**) 2007–2018, (**B**) 2007–2010, (**C**) 2011–2014, and (**D**) 2015–2018 in Belém, PA, Brazil.

In the spatial analysis, we considered 5985 (99.63%) cases since notifications were made without the home address for 22 cases. Figure 3 shows the spatial distribution of the HIV/AIDS sex-adjusted incidence rates for the 12 years of the study (Figure 3A) and for each quadrennium (Figure 3B–D). The highest incidences were found in the central and transition areas of the municipality.

**Figure 4:**
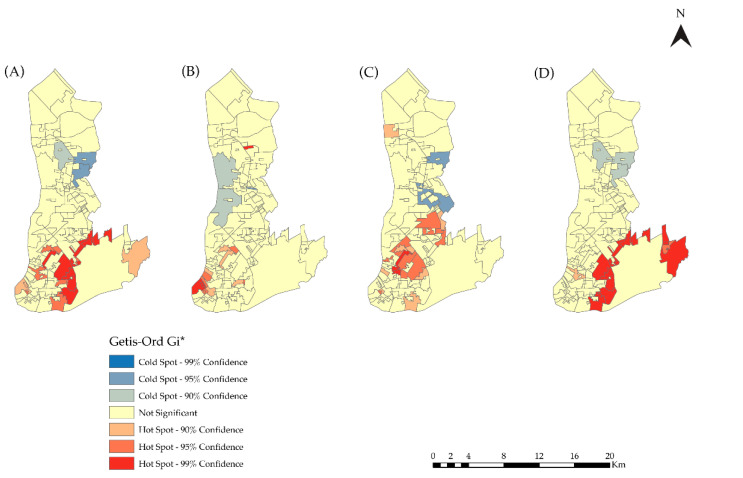
Map of Gi* local results. (**A**) 2007–2018, (**B**) 2007–2010, (**C**) 2011–2014, and (**D**) 2015–2018 in Belém, PA, Brazil.

The Getis–Ord General G analysis showed that the G indexes were statistically significant for the 12 years of the study and for the second and the third quadrenniums (2011–2014 and 2015–2018, respectively) (Table 2). However, the local index, Gi* local, showed clusters with hotspot and coldspot areas of statistical significance in all the analyzed periods (Figure 4). Hotspots were observed in the central and transition areas of Belém with a 99% or 95% confidence level. The coldspots were located in the expansion area of the city.

**Figure 5:**
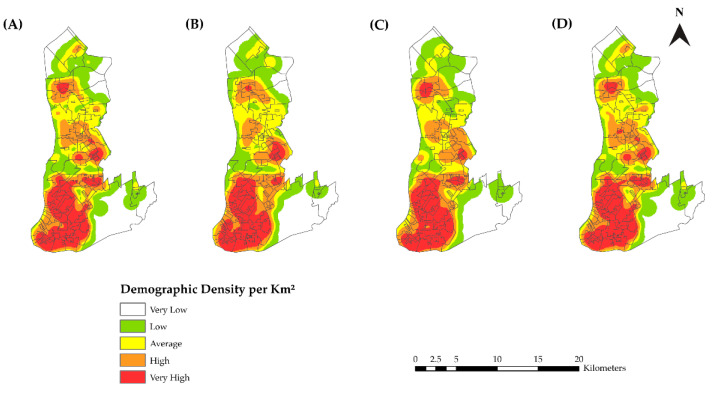
Map of the distribution of the density of new reported cases of HIV infections and AIDS. (**A**) 2007–2018, (**B**) 2007–2010, (**C**) 2011–2014, and (**D**) 2015–2018 in Belém, PA, Brazil.

Figure 5A shows the Kernel density analysis results for the period of the study. A higher concentration of new reported cases of HIV infections and AIDS was observed in the central area compared with the other areas. The analysis per quadrennium showed an expansion of the HIV epidemic from the central area toward the expansion area (Figure 5B–D).

**Figure 6:**
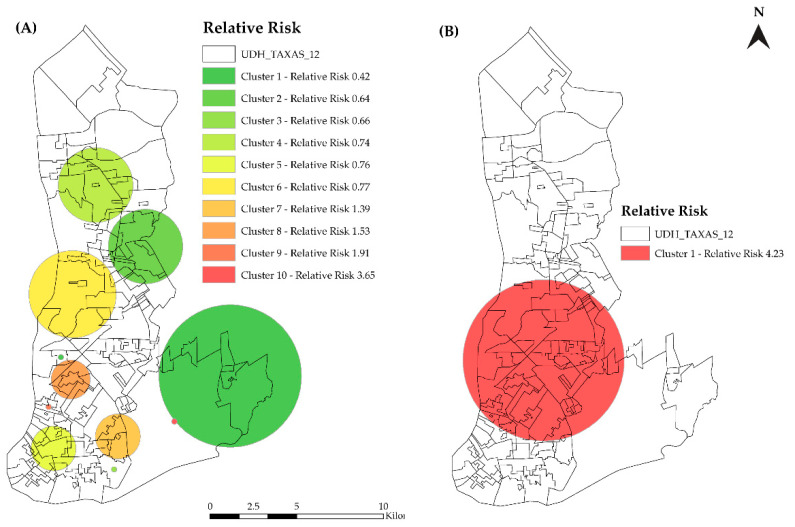
(**A**) Spatial clusters of new reported cases of HIV/AIDS. (**B**) Space–time cluster of new reported cases of HIV/AIDS in Belém, PA, Brazil (2007–2018).

Figure 6A,B shows the spatial and spatiotemporal risk for HIV/AIDS. Four areas were considered to have a spatial risk for HIV (cluster 7: RR = 1.39, CI 95% = 1.26–1.54, *p* = 0.000; cluster 8: RR = 1.53, CI 95% = 1.40–1.68, *p* = 0.001; cluster 9: RR = 1.91, CI 95% = 1.46–2.49, *p* = 0.013; cluster 10: RR = 3.65, CI 95% = 2.47–5.34, *p* = 0.004). The spatiotemporal risk area was identified as the period of 2017–2018, and the cluster consisted of 58 HDUs with a population of 1,988,264 inhabitants, 1708 HIV/AIDS new reported cases, and an incidence rate of 41.3 (×100,000 inhabitants) (RR = 4.24, CI 95% = 3.92–4.52, *p* = 0.003).

Table S2 shows Pearson’s correlation analysis between the sex-adjusted HIV/AIDS incidence rates and the independent variables. In the construction of the OLS, three variables with *p*-values ≤ 0.05 were used. Only the variable ‘Family Health Strategy (FHS) coverage’ was used in the GWR due to the smaller multicollinearity and AIC values (GWR: R² = 0.40; Adjusted R² = 0.38, AICc = 1313.7; OLS: R² = 0.27; Adjusted R² = 0.27, AIC = 1334.2). The analysis of the spatial autocorrelation of the residues of the GWR showed no spatial dependence (Moran I = 0.05, *p*-value = 0.184).

**Figure 7:**
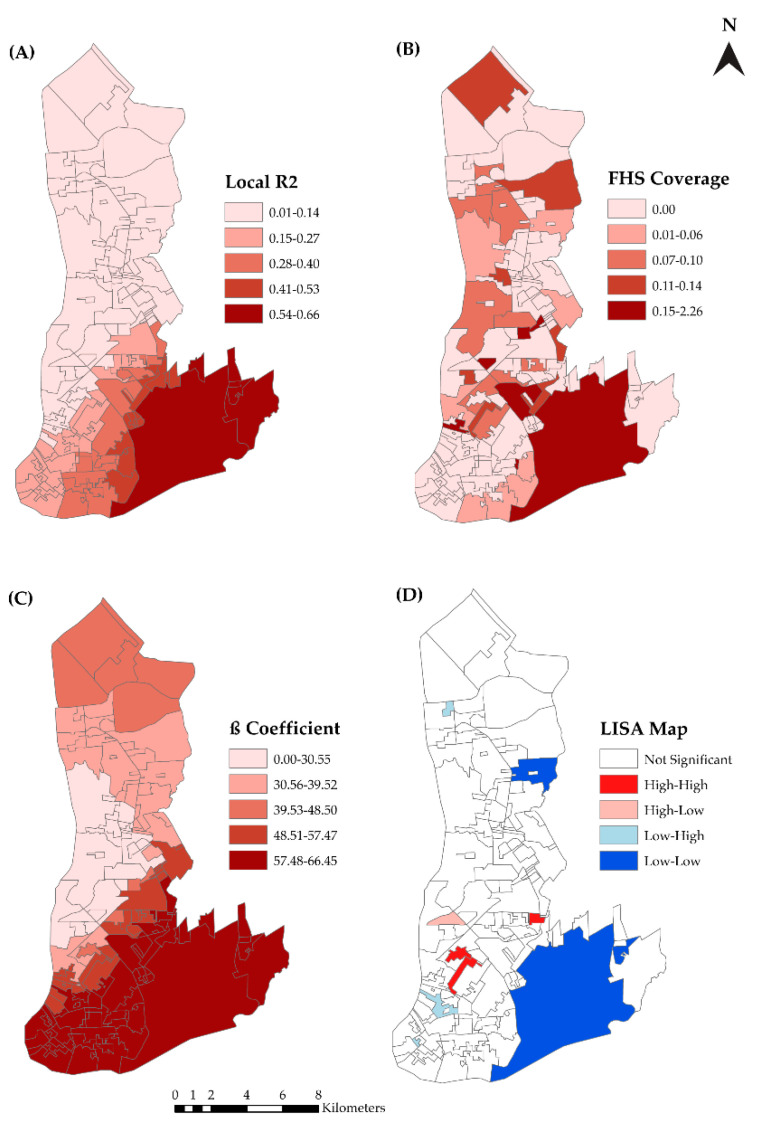
Spatial analysis of the social determinants of health and their association with HIV epidemic in Belém, PA, Brazil (2007–2018). (**A**) the local R^2^ value ranged from 0.01 to 0.66; (**B**) the distribution of FHS coverage in Belém. The β coefficient values indicated where the explanatory variable had the greatest influence on the dependent variable. The central area of Belém was where the FHS coverage had the strongest influence on the incidence of the HIV epidemic (**C**). (**D**) confirms the independence of the model residuals.

Figure 7 shows the GWR analysis maps. For this model, the local R² value ranged from 0.01 to 0.66 (Figure 7A). Figure 7B shows the distribution of FHS coverage in Belém. The β coefficient values indicated where the explanatory variable had the greatest influence on the dependent variable. The central area of Belém was where the FHS coverage had the strongest influence on the incidence of the HIV epidemic (Figure 7C). Figure 7D confirms the independence of the model residuals.

## 4. Discussion

The HIV epidemic in Belém had a stable trend from 2007 to October 2016. A peak in incidence was found in December 2016, after which irregular fluctuations occurred. The forecast model showed the stability and seasonality of the epidemic from 2019 to 2022, with higher rates from January to May compared with the rest of the year. The highest HIV/AIDS incidence rates were observed in the central area of Belém, with the epidemic growing in the transition and expansion areas. The spatial risk zones were located in the central area, whereas the spatiotemporal risk area was located in HDUs from all areas of the city and was temporally restricted between 2017 and 2018. The incidence rates were directly correlated with the FHS coverage. The β coefficient showed that the HIV/AIDS rates in the central area were more sensitive to changes in the FHS coverage than the transition and expansion areas.

Our results were different from those obtained in African countries, where the policy ‘Treatment for All’ has decreased the number of HIV/AIDS cases and the AIDS mortality rate [32]. However, this phenomenon is not only particular to Belém. In Libya, the intensity of HIV gradually decreased between 1993 and 2007, a period of 25 years. This period was followed by a subsequent increase in incidence, which peaked in 2017. Intravenous drug use and heterosexual activities were identified as the main predisposing factors [5]. In our temporal analysis, the noticed incidence rate peak in December 2016 was a reflection of a widely diffuse HIV testing campaign conducted in Belém in that month, which increased the HIV diagnosis. However, with the discontinuation of the campaign, the HIV/AIDS diagnosis rates decreased. This result shows the necessity of expanding the population accessibility to the HIV tests. In this sense, in 2012, the Brazilian Health Ministry decentralized the tests to the Primary Healthcare Network. However, some challenges still persist, such as unqualified health professionals for testing and diagnosis and insufficient number of tests for the assisted population [33].

Additionally, in Belém, the PrEP distribution and the HIV treatment health services are still centralized. Considering the low economical conditions of people from Belém, the decentralization of these services to the Primary Healthcare Network is essential to promote greater accessibility to these services. In Manaus, capital of Amazonas state, a study showed that the HIV treatment decentralization increased PLHIV satisfaction and ART adherence compared to those who assisted in centralized health centers [34,35].

In New York City, a study on the geographical distribution of Pre-Exposure Prophylaxis (PrEP)-dispensing facilities and on the relationships among their location, neighborhood characteristics, and HIV incidence found that providers of the prophylaxis were also located in neighborhoods with a high incidence of infection. A one-person-per-year increase in HIV incidence was associated with a 211 (per 100,000) increase in the density of PrEP dispensers [12].

The low level knowledge about HIV is another barrier to the elimination of the virus. In this direction, since 2007, the Brazilian Ministry of Health implemented the Health in School Program, in which the sexual and reproductive health education of young students is addressed. However, a study performed among young public high school students in Belém showed a low level of knowledge, risk behaviors toward the virus, and unawareness of the HIV testing and prevention services places [36].

In addition to the issues of health service coverage, the centralization of monitoring, and drug distribution, we also identified stigma and confidentiality as barriers to the implementation of the ‘Treatment for All’ policy in Belém, thus justifying the irregularity of the original data, as well as their unpredictability, given the high value of the MAPE [37]. On the contrary, in Zimbabwe, a study of time series ARIMA methods with HIV cases in people aged 15 years and older revealed an ‘almost’ constant mean over time, and after the expansion of access to treatment, new HIV infections are expected to decrease between 2019 and 2030, from approximately 31,321 to 20,071 new cases of HIV. The quality of this prediction showed very low measures of accuracy: On average, only 3.37% of the total number of new HIV infections are incorrect [38].

The expected seasonality may be attributed to the discontinuity of policies to combat HIV, especially prevention services, as in the indication of PrEP, which began to be offered in Belém from 2018. In a study conducted with men and women with a high likelihood of exposure to HIV, negative reactions were observed from professionals regarding PrEP, such as not believing in the safety or effectiveness of the method, attributing a higher degree of protection to condoms and other classic methods, and difficulties in dealing with reports of non-use of condoms concomitant with PrEP [39]. Additionally, associated with discontinued HIV prevention policies, the forecasted increase in the incidence rates from January to May reflect the end-of-year festivities in Belém, which increase people’s exposure to the virus and increase the HIV diagnosis in the subsequent months. In October, the largest catholic festivity of Pará, ‘Círio de Nazaré’, attracts millions of tourists to the city, and in December, people go to the beaches to celebrate the new year.

The areas in Belém that are hotspots for HIV/AIDS incidence rates, as well as the areas at risk of HIV/AIDS in the central area, have a high population density and numerous places that provide healthcare. Moreover, a study in Rio de Janeiro, Brazil, showed that the municipalities with higher HIV incidence rates among the elderly are the ones with the best economic situation and high accessibility to the healthcare system [10]. In China, a study showed that outbreaks among men who have sex with men were also located in areas with large populations and high economic growth [7].

The spatial–temporal risk cluster identified in Belém, in line with the evidence collected in Zimbabwe [38], may be associated with the success of strategies to combat the virus, such as the increased availability of rapid testing in Brazil between 2016 and 2017 [40]. Moreover, as a possible result of this success and as a corroboration of the findings of this study, Belém increased in the ranking among capital cities according to the composite index in the epidemiological bulletins of the disease in 2017, 2018, and 2019. Progressively, the capital of Pará moved from the third position to the first position in terms of the indicators of detection rates, mortality, and first CD4 count [41,42,43].

The HIV epidemic has spread to the expansion area of Belém; this may be associated with the characterization of this area as a peripheral area, where the population lives with precarious health and sports and leisure services [44]. In Pernambuco, Brazil, a study on users enrolled in the Specialized Outpatient Service from 2010 to 2014 revealed a modification in the profile of PLHIV cases, which were previously only concentrated in large urban centers. PLHIV cases are now also observed in regions with less urban development; the numbers can be explained by social factors, such as poverty, low education, and the deficiency of health services [45].

This study was limited by the ecological fallacy, indicating that the results cannot be considered at an individual level. The use of secondary data is also a limitation, as the quality of filled data may constitute an information bias in the investigation.

## 5. Conclusions

The incidence of HIV/AIDS in Belém peaked in December 2016 due to a widespread HIV testing action. The prediction model showed incidence seasonality from 2019 to 2022, which could be associated with the non-continuous HIV policies in the months of festivities in the city. The central area of Belém was the region most affected by the epidemic. The HIV epidemic progressed from the central area to transition and expanding areas. The largest spatial–temporal risk zones were formed by HDUs from central, peripheral, and transition areas from 2017 to 2018. The HIV/AIDS incidence rates were directly correlated with the coverage of FHS in the HDUs.

This paper provides data for health authorities to consider in combatting HIV in Belém. Significantly more than providing sexual health education in schools and communities, it is necessary to increase the population accessibility to diagnosis, preventive, and treatment healthcare places. The HIV prevention campaigns should be reinforced during the festivities months. Decentralizing testing, PrEP, PEP, and ART as well as expanding the coverage of FHS are necessary to ensure universal access to healthcare among Belém citizens.

## Figures and Tables

**Table 1:** Residuals and forecasting parameters of the STL + ARIMA model (2,1,2).

Test	*p*-Value
Kolmogorov–Smirnov	<0.001
F	0.712
Box–Pierce	0.808
	Model STL + ARIMA (2,1,2)
RMSE	0.64
MAE	0.35
MAPE	83.48

**Table 2:** Getis–Ord General G spatial association analysis of HIV infection and AIDS incidence rates in Belém, PA, Brazil (2007–2018).

Incidence Rate	Observed General G	Z-Score	*p*-Value
2007–2018	0.041	3.464	0.000
2007–2010	0.036	0.642	0.524
2011–2014	0.046	4.157	0.008
2015–2018	0.043	3.404	0.001

## Data Availability

The datasets analyzed during the current study are not publicly available due restrictions which are applied to the availability of these data. All the data in this study were used under license.

## Supplementary Materials

The following supporting information can be downloaded at https://www.mdpi.com/article/10.3390/tropicalmed7090225/s1. Table S1: Epidemiological profile of new reported cases of HIV/AIDS, Belém, Pará, Brazil (2007–2018); Table S2: Analysis of the correlation between socioeconomic indicators and the HIV/AIDS incidence rate, Belém, Pará, Brazil (2007–2018).

## References

[1] Joint United Nations Programme on HIV and AIDS (2020). UNAIDS Report on the Global AIDS Epidemic Shows that 2020 Targets Will not Be Met because of Deeply Unequal Success; COVID-19 Risks Blowing HIV Progress Way off Course. https://www.unaids.org/en/resources/presscentre/pressreleaseandstatementarchive/2020/july/20200706_global-aids-report.

[2] Pan American Health Organization (2020). New Cases of HIV Infection Increased by More than 20% in Latin America in the Last Decade. https://www.paho.org/pt/noticias/30-11-2020-novos-casos-infeccao-por-hiv-aumentaram-mais-20-na-america-latina-na-ultima.

[3] Ministry of Health of Brazil, Secretariat of Health Surveillance, Department of Chronic Conditions Diseases and Sexually Transmitted Infections (2020). Epidemiological Report HIV/AIDS 2020.

[4] de Holanda E.R., Galvão M.T.G., Pedrosa N.L., Paiva S.D.S., De Almeida R.L.F. (2015). Spatial analysis of infection by the human immunodeficiency virus among pregnant women1. Rev. Lat. Am. Enferm..

[5] Daw M.A., Daw A.M., Sifennasr N.E.M., Draha A.M., Daw A.A., Daw A.A., Ahmed M.O., Mokhtar E.S., El-Bouzedi A.H., Daw I.M. (2019). Spatiotemporal analysis and epidemiological characterization of the human immunodeficiency virus (HIV) in Libya within a twenty five year period: 1993–2017. AIDS Res. Ther..

[6] Falavina L.P., Lentsck M.H., Mathias T.A.D.F. (2019). Tendência e distribuição espacial de doenças infecciosas em gestantes no estado do Paraná-Brasil. Rev. Lat. Am. Enferm..

[7] Qin Q., Guo W., Tang W., Mahapatra T., Wang L., Zhang N., Ding Z., Cai C., Cui Y., Sun J. (2017). Spatial Analysis of the Human Immunodeficiency Virus Epidemic among Men Who Have Sex with Men in China, 2006–2015. Clin. Infect. Dis..

[8] Wand H., Ramjee G. (2015). Spatial clustering of “measured” and “unmeasured” risk factors for HIV infections in hyper-endemic communities in KwaZulu-Natal, South Africa: Results from geoadditive models. AIDS Care.

[9] Zhang X.Y., Huang T., Feng Y.B., Li M., Chen F.F., Li Y.G., Jin S.S., Bu K., Wang L. (2015). Characteristics of the HIV/AIDS Epidemic in Women Aged 15–49 Years from 2005 to 2012 in China. Biomed. Environ. Sci..

[10] Rodrigues N.C.P., De Almeida A.S., Braga J.U., O’Dwyer G., Junior P.C.A., Daumas R.P., Lino V.T.S., Andrade M.K.D.N., Monteiro D.L.M., Barros M.B.D.L. (2015). Spatial dynamics of AIDS incidence in the elderly in Rio de Janeiro, Brazil, 1997–2011. Cad. Saúde Pública..

[11] Wang Y., Yang Y., Shi X., Mao S., Shi N., Hui X. (2016). The spatial distribution pattern of human immunodeficiency virus/acquired immune deficiency syndrome in China. Geospat. Health.

[12] Kim B., Callander D., DiClemente R., Trinh-Shevrin C., Thorpe L.E., Duncan D.T. (2019). Location of Pre-exposure Prophylaxis Services Across New York City Neighborhoods: Do Neighborhood Socio-demographic Characteristics and HIV Incidence Matter?. AIDS Behav..

[13] Chen M., Ma Y., Chen H., Dai J., Luo H., Yang C., Dong L., Jin X., Yang M., Yang L. (2019). Spatial clusters of HIV-1 genotypes in a recently infected population in Yunnan, China. BMC Infect. Dis..

[14] Chen M., Ma Y., Chen H., Dai J., Luo H., Yang C., Dong L., Jin X., Yang M., Yang L. (2019). Demographic characteristics and spatial clusters of recent HIV-1 infections among newly diagnosed HIV-1 cases in Yunnan, China, 2015. BMC Public Health.

[15] Gelaw Y.A., Magalhães R.J.S., Assefa Y., Williams G. (2019). Spatial clustering and socio-demographic determinants of HIV infection in Ethiopia, 2015–2017. Int. J. Infect. Dis..

[16] Ramjee G., Sartorius B., Morris N., Wand H., Reddy T., Yssel J.D., Tanser F. (2019). A decade of sustained geographic spread of HIV infections among women in Durban, South Africa. BMC Infect. Dis..

[17] Merzouki A., Styles A., Estill J., Orel E., Baranczuk Z., Petrie K., Keiser O. (2020). Identifying groups of people with similar sociobehavioural characteristics in Malawi to inform HIV interventions: A latent class analysis. J. Int. AIDS Soc..

[18] Brazilian Institute of Geography and Statistics (2010). 2010 Census. https://ww2.ibge.gov.br/home/.

[19] Municipal Health Secretary of Belém (2017). Municipal Health Plan (MHP) 2018–2021.

[20] United Nations Development Programme (2013). Atlas of Human Development of Brazil. https://atlasbrasil.org.br/2013/.

[21] Institute for Applied Economic Research (2015). Atlas of Social Vulnerability in Brazilian Municipalities. https://ivs.ipea.gov.br/images/publicacoes/Ivs/publicacao_atlas_ivs.pdf.

[22] Bailey T.C., Gatrell A.C. (1995). Interactive Spatial Data Analysis.

[23] Belém (2016). Belém Urban Mobility Plan.

[24] Box G.E.P., Jenkins G.M., Reinsel G.C. (2015). Time Series Analysis: Forecasting and Control.

[25] Hyndman R.J., Athanasopoulos G. (2018). Forecasting: Principles and Practice.

[26] Rothman K.J., Greenland S., Lash T.L. (2011). Modern Epidemiology.

[27] Ministry of Health of Brazil, Secretariat of Health Surveillance (2007). Oswaldo Cruz Foundation Introduction to Spatial Statistics for Public Health.

[28] Fusco A.P.B., Arcêncio R.A., Yamamura M., Palha P.F., dos Reis A.A., Alecrim T.F.D.A., Protti S.T. (2017). Spatial distribution of tuberculosis in a municipality in the interior of São Paulo, 2008–2013. Rev. Lat. Am. Enferm..

[29] Kulldorff M., Nagarwalla N. (1995). Spatial disease clusters: Detection and inference. Stat. Med..

[30] Wagner M.B., Cellegari-Jacques S.M. (1998). Measures of association in epidemiological studies: Relative risk and odds ratio. J. Pediatr..

[31] De Assis I.S., Arcoverde M., Ramos A.C.V., Alves L.S., Berra T.Z., Arroyo L.H., De Queiroz A.A.R., Dos Santos D.T., Belchior A.D.S., Alves J.D. (2018). Social determinants, their relationship with leprosy risk and temporal trends in a tri-border region in Latin America. PLoS Negl. Trop. Dis..

[32] Joint United Nations Programme on HIV and AIDS (2017). China Focuses on Strengthening HIV Prevention. https://unaids.org.br/2017/11/china-se-concentra-no-fortalecimento-da-prevencao-do-hiv/.

[33] Ministry of Health of Brazil (2012). Ordinance No. 77, of 12 January 2012. Provides for Rapid Tests, in Primary Care, for the Detection of HIV and Syphilis, as well as Rapid Tests for Other Diseases, within the Scope of Prenatal Care for Pregnant Women and Their Sexual Partners.

[34] Leon C., Koosed T., Philibert B., Raposo C., Benzaken A.S. (2019). HIV/AIDS health services in Manaus, Brazil: Patient perception of quality and its influence on adherence to antiretroviral treatment. BMC Health Serv. Res..

[35] Castro R.R., De Oliveira S.S., Pereira I.R.B.D.O., Dos Santos W.N., Fernandes S.F., Da Silva R.A.R. (2019). Construct validation: Coping with HIV/AIDS in Primary Health Care. Rev. Bras. Enferm..

[36] De Lima M.S., Raniere J.C., Paes C.J.O., Gonçalves L.H.T., Cunha C.L.F., Ferreira G.R.O.N., Botelho E.P. (2020). The association between knowledge about HIV and risk factors in young Amazon people. Rev. Bras. Enferm..

[37] Moreno J.J.M., Pol A.P., Abad A.S. (2013). Using the R-MAPE index as a resistant measure of forecast accuracy. Psicothema.

[38] Nyoni S.P., Nyoni T. (2020). Adults newly infected with HIV in Zimbabwe: A box-jenkins ARIMA approach. Int. J. Innov. Eng. Res. Technol..

[39] Zucchi E.M., Grangeiro A., Ferraz D., Pinheiro T.F., Alencar T., Ferguson L., Estevam D.L., Munhoz R. (2018). From evidence to action: Challenges for the Brazilian National Health System to offer HIV pre-exposure prophylaxis (PrEP) to the most vulnerable people. Cad. Saúde Pública..

[40] Ministry of Health of Brazil (2017). Ministry of Health Records Increases in HIV Cases in 2016.

[41] Ministry of Health of Brazil, Secretariat of Health Surveillance, Department of Chronic Conditions Diseases and Sexually Transmitted Infections (2017). Epidemiological Report HIV/AIDS 2017.

[42] Ministry of Health of Brazil, Secretariat of Health Surveillance, Department of Chronic Conditions Diseases and Sexually Transmitted Infections (2018). Epidemiological Report HIV/AIDS 2018.

[43] Ministry of Health of Brazil, Secretariat of Health Surveillance, Department of Chronic Conditions Diseases and Sexually Transmitted Infections (2019). Epidemiological Report HIV/AIDS 2019.

[44] Vieira D.C.D.M., Rodrigues J.C., Rodrigues J.C. (2018). Mapeamento e análise de desigualdades socioespaciais: Abordagem interpretativa a partir da cidade de Belém, Pará. Geosaberes.

[45] Santos N.T.N., Silva S.P.C., Fernandes F.E.C.V., Santana L.D., Silva T.I.M. (2019). Epidemiological profile of HIV/AIDS cases registered in a Specialized Outpatient Service. Rev. Gest. Saúde.

