# Supplementary material for: The Temporal and Spatial Epidemiology Employed in the Elimination of the HIV Epidemic in the Largest Capital of the Brazilian Rainforest
Source: Trop Med Infect Dis. 2022 Sep 2;7(9):225. doi: 10.3390/tropicalmed7090225 (PMC9505481; doi:10.3390/tropicalmed7090225)
Supplement: Supplementary file 1 [file tropicalmed-07-00225-s001.zip › tropicalmed-1865895-Table S1.pdf]

Tropical Medicine and Infectious Disease

Article

The Temporal and Spatial Epidemiology Employed in the Elimination of the HIV Epidemic in the Largest Capital of the Brazilian Rainforest

Bruna Rafaela Leite Dias [1,*], Taymara Barbosa Rodrigues [1], Dulce Gomes [2], Ricardo Alexandre Arcêncio [3], Elucir Gir [4], Glenda Roberta Oliveira Naiff Ferreira [1], Sandra Helena Isse Polaro [1], Eliã Pinheiro Botelho [1]

1 Graduate Nursing Program; Federal University of Pará, Belém 66075-110, Brazil
2 Department of Mathematics, Luís António Verney College, University of Évora, 7000-671 Évora, Portugal
3 Department of Maternal-Infant and Public Health Nursing, College of Nursing at Ribeirão Preto, University of São Paulo, 14040-902 Ribeirão Preto, Brazil
4 College of Nursing at Ribeirão Preto, University of São Paulo, 14040-902 Ribeirão Preto, Brazil
* Correspondence: brunarafaela_jm@hotmail.com

Table S1. Epidemiological profile of new reported cases of HIV/AIDS. Belém/Pará, Brazil (2007 - 2018).

Variables	n	%
**Age (Years)**		
11-14	14	0,2
15-29	2184	36,3
30-49	3059	50,9
50+	750	12,5
**Sex**		
Female	1760	29,3
Male	4245	70,7
Unanswered	2	0,0
**Race/skin color**		
Black	417	6,9
Brown	4180	69,6
Indigenous	13	0,2
White	686	11,4
Yellow	20	0,3
Unanswered	691	11,5
**Schooling**		
Elementary School	1763	29,3
High School	1936	32,2
Higher Education	796	13,3
Illiterate	37	0,6
Unanswered	1475	24,6
**Exposure category**		
Female/Bisexual/Haemophilic	0	0,0
Female/Bisexual/Intravenous drug user	1	0,02

Female/Bissexual	16	0,26
Haemophilic	1	0,0
Heterosexual	3149	52,4
Heterosexual/Haemophilic	2	0,0
Heterosexual/Intravenous drug user	90	1,5
Homosexual	1721	28,6
Homosexual/Haemophilic	1	0,0
Homosexual/Intravenous drug user	19	0,3
Intravenous drug user	17	0,3
Male/Bisexual/Haemophilic	1	0,0
Male/Bisexual/Intravenous drug user	5	0,08
Male/Bissexual	408	6,83
Vertical transmission	62	1,0
Unanswered	514	8,6
