# Supplementary material for: The Temporal and Spatial Epidemiology Employed in the Elimination of the HIV Epidemic in the Largest Capital of the Brazilian Rainforest
Source: Trop Med Infect Dis. 2022 Sep 2;7(9):225. doi: 10.3390/tropicalmed7090225 (PMC9505481; doi:10.3390/tropicalmed7090225)
Supplement: Supplementary file 1 [file tropicalmed-07-00225-s001.zip › tropicalmed-1865895-Table S2.pdf]

Tropical Medicine and Infectious Disease

Article

The Temporal and Spatial Epidemiology Employed in the Elimination of the HIV Epidemic in the Largest Capital of the Brazilian Rainforest

Bruna Rafaela Leite Dias [1,*], Taymara Barbosa Rodrigues [1], Dulce Gomes [2], Ricardo Alexandre Arcêncio [3], Elucir Gir [4], Glenda Roberta Oliveira Naiff Ferreira [1], Sandra Helena Isse Polaro [1], Eliã Pinheiro Botelho [1]

[1] Graduate Nursing Program; Federal University of Pará, Belém 66075-110, Brazil
[2] Department of Mathematics, Luís António Verney College, University of Évora, 7000-671 Évora, Portugal
[3] Department of Maternal-Infant and Public Health Nursing, College of Nursing at Ribeirão Preto, University of São Paulo, 14040-902 Ribeirão Preto, Brazil
[4] College of Nursing at Ribeirão Preto, University of São Paulo, 14040-902 Ribeirão Preto, Brazil
* Correspondence: brunarafaela_jm@hotmail.com

Table S2. Analysis of correlation between socioeconomic indicators and HIV/AIDS incidence rate. Belém/Pará, Brazil (2007-2018).

Variable	Collinearity	p-value
% of population living with more than 2 people in the house	0.002	0,978
% of population living in houses with tap water	0.224	0.006
% of population living in house with bathroom and tap water	0.080	0.331
% of population living in urban households with garbage collection service	0.046	0,572
% of population living in houses having electric power	0.061	0,456
% of people in houses having inadequate water supply and sewage disposal	-0.076	0,351
% of people living in homes with walls other than masonry or wood shavings	-0.077	0,347
18 years old and over people illiteracy rate	-0.043	0,597
% 18 years and over people with complete elementary schooling level	0.032	0,695
% of 25 years old and over people with complete elementary schooling level	0.032	0,697
% 18 years old and over people with complete high schooling level	0.022	0,787
% of 25 years old and over people with complete university schooling level	-0.054	0,509

% of employed people with elementary schooling	0.057	0,487
% of employed people with high schooling	0.035	0,666
% of employed people with university schooling	-0.048	0,559
IDHM	0.019	0,821
Gini Index	0.127	0,119
Theil Index – L	0.099	0,226
IVS	-0.033	0,690
Family Health Strategy Coverage	0.527	0,000
% of extremely poor people	-0.080	0,390
% of poor people	-0.059	0,469
% of people vulnerable to poverty	-0.039	0,631
% of total rental income appropriated by the 10% of the population with the highest per capita household income	0.173	0,033
% of total rental income appropriated by the 20% of the population with lowest per capita household	-0.041	0,619
% of total rental income appropriated by the 20% of the population with the highest per capita household income	0.154	0,059
Population average per capita income	-0.081	0,321
Average per capita household income of the quintile of the poorest population	-0.088	0,281
Average per capita household income of the second quintile of the poorest population	-0.095	0,246
Average per capita household income of the third quintile of the poorest population	-0.102	0,213
Average per capita household income of the of the fourth quintile of the poorest population	-0.101	0,218
Average per capita household income of the poorest population	0.050	0,589
Average per capita household income of poor people	0.070	0,392
Average per capita household income of people vulnerable to poverty	0.011	0,893
% of 18 years old and over self-employed people	-0.028	0,737
% of 18 years old and over hired people	0.157	0,054

% of employees with household income up to 1 minimum monthly wage	-0.061	0,455
% of employees with monthly income up to 5 minimum monthly wages	0.069	0,399
Job activity rate for 18 years old and over people	-0.152	0,063
